# Toward Kilogram-Scale Peroxygenase-Catalyzed Oxyfunctionalization of Cyclohexane

**DOI:** 10.1021/acs.oprd.3c00135

**Published:** 2023-06-09

**Authors:** Thomas Hilberath, Remco van Oosten, Juliet Victoria, Hugo Brasselet, Miguel Alcalde, John M. Woodley, Frank Hollmann

**Affiliations:** † Department of Biotechnology, Delft University of Technology, van der Maasweg 9, 2629HZ Delft, The Netherlands; ‡ Department of Chemical and Biochemical Engineering, Technical University of Denmark, 2800 Kgs. Lyngby, Denmark; § Atlant. Innov., Koornmarkt 52, 2611 EH Delft, The Netherlands; ∥ Department of Biocatalysis, Institute of Catalysis, CSIC, 28049 Madrid, Spain

**Keywords:** biocatalysis, peroxygenase, oxyfunctionalization, cyclohexane, upscaling, bulk chemical

## Abstract

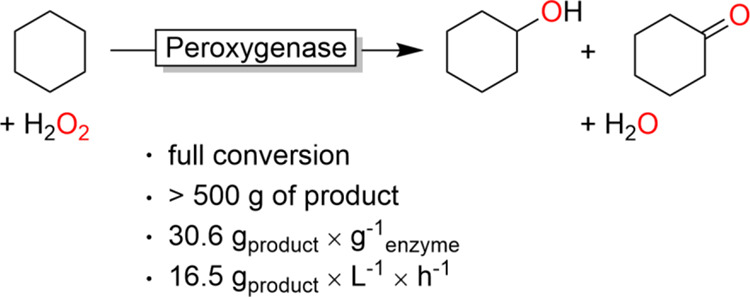

Mol-scale oxyfunctionalization of cyclohexane to cyclohexanol/cyclohexanone (KA-oil) using an unspecific peroxygenase is reported. Using *Aae*UPO from *Agrocybe aegerita* and simple H_2_O_2_ as an oxidant, cyclohexanol concentrations of more than 300 mM (>60% yield) at attractive productivities (157 mM h^–1^, approx. 15 g L^–1^ h^–1^) were achieved. Current limitations of the proposed biooxidation system have been identified paving the way for future improvements and implementation.

## Introduction

Biocatalysis is increasingly considered as an alternative to traditional chemical methodologies. In particular, the high selectivity of many enzymes is especially valued for the synthesis of chiral, value-added products.^1−3^ As a consequence, the overwhelming majority of biocatalytic processes in the chemical industry deals with the synthesis of fine or specialty chemicals or pharmaceutical intermediates. Bulk chemical applications such as the synthesis of acrylamide are scarce.^4^

**Scheme 1:**
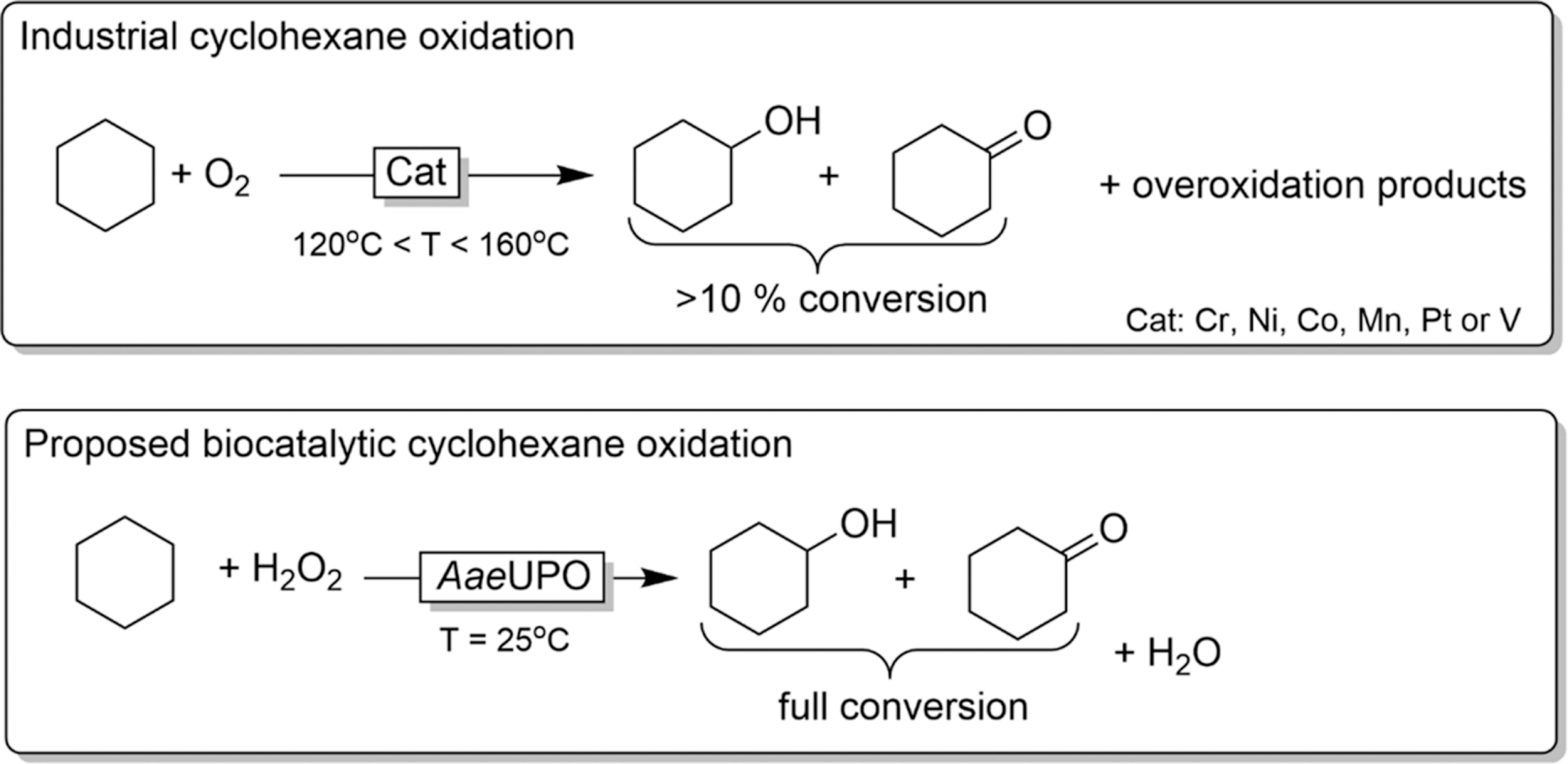
Oxidation of Cyclohexane Outlined are the established aerobic oxidation procedures (upper) and the proposed biocatalytic alternative (lower).

The biocatalytic oxyfunctionalization of cycloalkanes, for example, is occasionally addressed in the literature^5−9^ but so far has not been considered as an alternative to existing industrial practice. Especially in the case of this transformation, high chemoselectivity would be highly desirable.^10^ The main issue for the chemical oxidation of cyclohexane lies with the increasing reactivity of the oxidation products. In other words, the rate of overoxidation of products is faster than the rate of desired oxidation of starting materials, thereby making isolation of intermediate products such as cyclohexanol or cyclohexanone challenging. Today, the technically implemented solution to this problem is to limit conversions to less than 10% and thereby minimize reagent loss in undesired overoxidation products (Scheme 1).^10^ Obviously, the unreacted starting material is recycled, which however also adds complexity to the production system.

Selective enzyme catalysts may represent a solution to this selectivity issue. In the past, especially cytochrome P450 monooxygenases (P450 MOs)^11,12^ have been considered as catalysts for the selective oxyfunctionalization of cycloalkanes.^13−20^ Though excellent results with full conversion and high selectivity have been achieved, the space time yields tend to be low, in the range of a few millimolar product formations per hour and low final product titers generally in the range of 5–10 mM.^21^ Next, the complex molecular architecture of many P450 MOs^22^ and also their dependency on molecular oxygen challenge their practical application at scale.^23−25^ So-called unspecific peroxygenases (UPOs, E.C. 1.11.2.)^26,27^ also catalyze the oxyfunctionalization of (cyclo)alkanes at high selectivity^28^ but at the same time only need hydrogen peroxide as the stoichiometric oxidant instead of the complex electron transfer chain to reductively activate molecular oxygen. Particularly, the UPO from *Agrocybe aegerita* (*Aae*UPO, PaDa-I variant)^29,30^ is an attractive biocatalyst for the oxyfunctionalization of, for example, cyclohexane.

Previously, we^31^ and the group of Hofrichter^28^ reported the selective, peroxygenase-catalyzed oxidation of cyclohexane yielding only cyclohexanol and cyclohexanone [i.e., ketone-alcohol (KA) oil] as products. However, in these studies, the substrate loading and consequently the product concentrations were in the lower millimolar (μmol) range and thus far too low for any preparative application.

Given the extraordinary stability and activity of *Aae*UPO,^32^ we set out to evaluate whether this enzyme may enable multi-mol-scale synthesis of KA-oil (Scheme 1).

## Materials and Methods

### Preparation of the Recombinant UPO from *Agrocybe aegerita* (*Aae*UPO, PaDa-I)

The biocatalyst (expression-engineered variant of the peroxygenase from *Agrocybe aegerita*, *Aae*UPO PaDa-I mutant) originated from a 2500 L pilot-scale cultivation of recombinant *Pichia pastoris* X-33.^33^ The concentrated supernatant was lyophilized at 0.1 mbar and −28 °C using a Christ Alpha 2–4 lyophilizer (Martin Christ Gefriertrocknungsanlagen GmbH, Osterode am Harz, Germany). For the 11 L reactions, 536 g of lyophilized enzyme with a total *Aae*UPO-amount of 456 μmol (0.85 μmol_*Aae*UPO_ g^–1^_lyophilisate_) was used.

### CO-Difference Spectra

*Aae*UPO concentrations were determined from carbon monoxide (CO)-difference spectra using the extinction coefficient at 445 nm of ε_445_ = 107 mM^–1^ cm^–1^.^34^ 950 μL of protein sample, diluted in 100 mM KPi-buffer, was filled into plastic cuvettes, placed in a photometer, and blank recorded (base subtraction). After zeroing, the sample was exposed to CO for a few seconds. Next, 50 μL of 1 M sodium dithionite stock solution was added, and a difference spectrum between 400 and 500 nm was recorded. The measurements were continued until a constant absorption maximum was obtained.

### H_2_O_2_ Quantification Assay

The concentration of H_2_O_2_ in the reactor was measured at different time points using a Pierce quantitative peroxide assay kit (catalog number 232802, Thermo Scientific Pierce, Rockford, IL, USA). The working reagent (WR) was prepared by mixing 100 μL of reagent A with 10 mL of reagent B as described in the kit. Samples were withdrawn every 15 min from the reactor for H_2_O_2_ analysis. Two dilutions were prepared for each sample, and the analysis of each dilution was performed in duplicate. 20 μL of sample was incubated with 200 μL of premixed WR in a 96-well plate and incubated at 25 °C for 15 mins.

The H_2_O_2_ concentration was determined by measuring the absorbance at 240 nm using a molar extinction coefficient of 43.6 M^–1^ cm^–1^.^35^ A standard curve was made with H_2_O_2_ concentrations ranging from 0 to 130 μM. The absorbance of standards and samples was measured at 595 nm using a microplate reader (SPECTRO star Nano; BMG LABTECH, Germany). The slope of the standard curve was used for quantification of the H_2_O_2_ concentration.

### 100 mL Scale Reactions

The *Aae*UPO-mediated hydroxylation of cyclohexane on a 100 mL scale was performed in a SYSTAG jacketed lab reactor (250 mL operational volume) at 25 °C and 300 rpm mixing speed. 100 mL of reaction solution contained 100 mM KPi buffer (pH 6), 50 vol % acetonitrile, 10–20 μM r*Aae*UPO (concentrated supernatant), and 500 mM cyclohexane. H_2_O_2_ solutions were freshly prepared prior to the experiment and continuously fed from a 4.5 M or 50 wt % stock solution with a H_2_O_2_-dosing rate of 50 and 200 mM h^–1^, respectively. The amount of added H_2_O_2_ per hour was kept constant at 1.2 g which corresponds roughly to 1.1 mL. The reaction was monitored for up to 48 h. At different time points, samples from the aqueous phase were withdrawn, extracted with 500 μL of ethyl acetate containing 5 mM of the internal standard *n*-dodecane (IS), and analyzed via achiral GC. Reaction mixtures were also qualitatively analyzed for H_2_O_2_ accumulation by color change using Quantofix peroxide 100 test strips (Macherey-Nagel, Düren, Germany).

### *Aae*UPO-Mediated Oxidation at 11 L Scale under Preparative Scale Conditions

**Figure 1:**
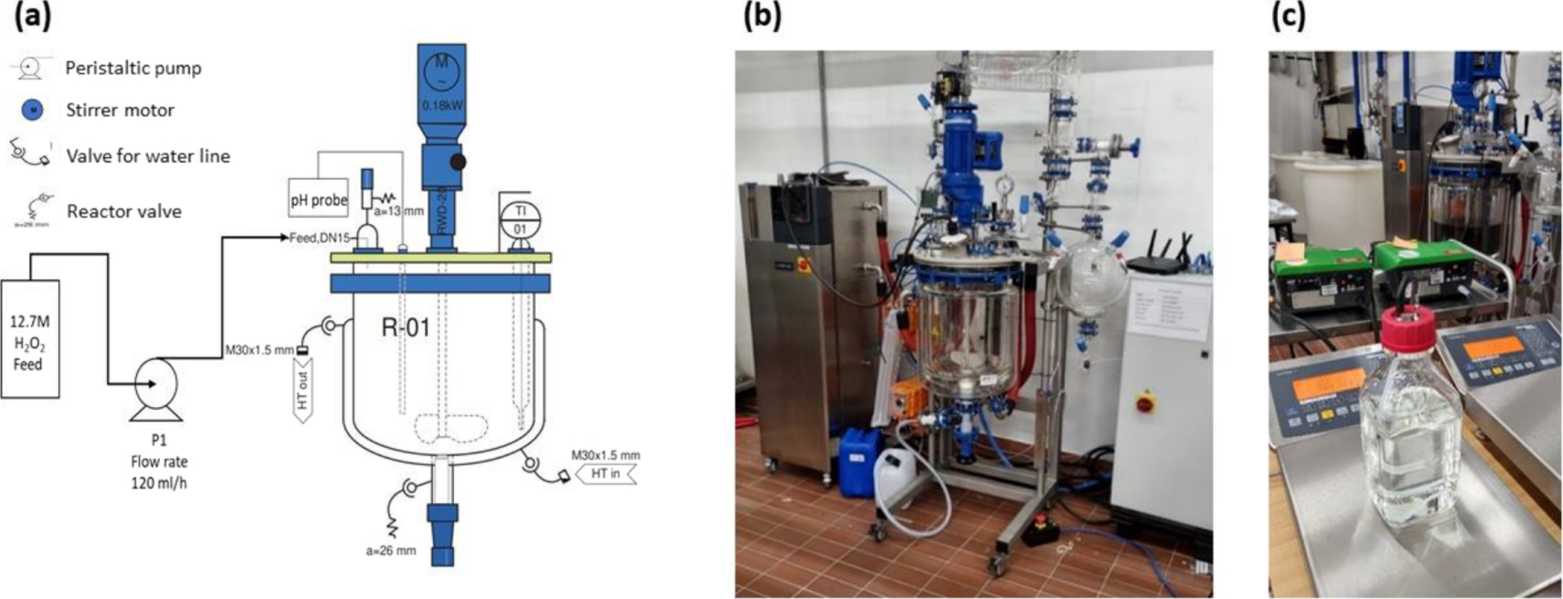
35 L-reactor used for the reaction on 10 L scale. (a) Process flow diagram (T1: temperature sensor, pH 01: pH sensor and display, P1: pump, HT in/out: hot water in/out, R-01: reactor). (b) Photograph of the reactor setup. (c) Photograph of the pump setup.

The UPO-mediated oxidation of cyclohexane was upscaled to a 11 L scale in a 35 L jacketed glass reactor (Figure 1). In total, two runs were performed.

#### Fed-Batch 1

258 g of lyophilized *Aae*UPO (PaDa-I variant) was dissolved in a total volume of around 5 L of 100 mM KPi, pH 6 and added to a 35 L jacketed glass reactor. Afterward, 5.5 L of acetonitrile was pumped at 0.5 L per min using a Watson Marlow peristaltic pump 503 S (Watson-Marlow, Falmouth, UK). The agitation speed was set at 225 rpm to maintain the same power input of 2 W/L as the 100 mL batches. Then, 600 mL of cyclohexane was added using the same pump (total volume 10.7 L). The reaction was started by pumping H_2_O_2_ from a 12.75 M solution with a flow rate of 120 mL/h using a Watson-Marlow peristaltic pump 520 S (Watson-Marlow, Falmouth, UK). Every 15 min, a 5 mL sample was taken and analyzed for H_2_O_2_ concentration via a photometric assay. The product concentration was analyzed every hour for the first 2 h and subsequently every 30 min via GC-FID. The reaction was stopped after 3.75 h.

#### Fed-Batch 2

278 g of lyophilized *Aae*UPO (PaDa-I variant) was dissolved in a total volume of around 5 L of 100 mM KPi, pH 6 and placed in 35 L jacketed glass reactor. Afterward, 5.5 L of acetonitrile was pumped at 0.5 L per min using a Watson Marlow peristaltic pump 503 S (Watson-Marlow, Falmouth, UK). The agitation speed was set at 225 rpm to maintain the same power input of 2 W/L as the 100 mL batches. Then, 630 mL of cyclohexane was added using the same pump (total volume 10.7 L). The reaction was started by pumping H_2_O_2_ from a 12.75 M solution with a flow rate of 120 mL/h. Every 15 min, a 5 mL sample was taken and analyzed for H_2_O_2_ concentration via a photometric assay. The product concentration was analyzed every hour for the first 2 h and subsequently every 30 min via GC-FID. The reaction was stopped after 3.75 h.

### Product Analysis

For GC analysis, a Shimadzu GC-2010 plus/FID equipped with an Agilent CP-Wax 52GB column (50 m × 0.53 mm × 2.0 μm) with N_2_ as the carrier gas was used. The temperature gradient was described in key points as follows: (Split 10): 90 °C hold 3 min, 10 °C/min to 180 °C hold 1 min, 30 °C/min to 230 °C hold 1 min, retention times: 10.4 min cyclohexanol, 9.1 min cyclohexanone, 6.7 min *n*-dodecane (IS).

## Results

To test the feasibility of the proposed *Aae*UPO-catalyzed oxidation of cyclohexane, we used a previously reported batch of *Aae*UPO produced at pilot scale.^33^ We envisioned an initial cyclohexane concentration of 0.5 M. Since *Aae*UPO exhibits an exceptional stability toward acetonitrile,^32^ we decided using acetonitrile (50% vol/vol) as the cosolvent to improve the solubility of the reagents. H_2_O_2_ was added continuously (fed-batch mode) from a concentrated stock solution (Table 1). We deem potential safety issues to be low (at least in the small-scale experiments presented here) as the H_2_O_2_ is constantly consumed by the enzymatic reaction.

**Table 1:** Influence of H_2_O_2_ Feeding Rate and Enzyme Concentration on a 100 mL Scale^a^

experiment	[*Aae*UPO] [μM]	[cyclohexane] [mM]	H_2_O_2_-feeding rate [mM h^–1^]	time [h]	total product concentration [mM]	productivity [g L^–1^ h^–1^]	turnover number (TN = mol_Product_ × mol_r*Aae*UPO_^–1^)
1	10	500	50	24	70.7	0.3	7000
2	20	500	50	24	390	1.6	19,500
3	20	500	200	4	373	9.3	18,650
4	20	3 × 500 (0, 6, 28 h)	20	24	269	1.1	13,450

a Reaction conditions: [substrate] = 500 mM, [KPi, pH 6] = 100 mM, [ACN] = 50 vol %, [*Aae*UPO] = 10–20 μM, [H_2_O_2_-feeding rate] = 20–200 mM h^–1^ (4–17.6 M aqueous stock solution) starting volume 100 mL, 25 °C, 300 rpm, duplicate measurements. The detailed time courses are listed in the Supporting Information (Figures S1–S4).

In the first experiment, we used 10 μM (ca. 0.45 g L^–1^) *Aae*UPO and a H_2_O_2_ feed rate of 50 mM h^–1^ (Table 1, entry 1, Figure S1). The initial product formation rate was approx. 37 mM h^–1^ corresponding to 74% of the nominal H_2_O_2_ addition rate. However, the reaction rate dropped significantly over time and after approx. 5 h essentially ceased. This was accompanied by the accumulation of H_2_O_2_ in the reaction mixture. As a result, only 70 mM cyclohexanol: cyclohexanone (11.2:1) were found after 24 h. We reasoned that the H_2_O_2_ addition rate may have exceeded the catalytic activity of *Aae*UPO for cyclohexane hydroxylation resulting in a steady increase in the H_2_O_2_ concentration. The accumulation of H_2_O_2_ inactivates the enzyme, decreasing further the catalytic rate and thereby increasing the rate of accumulation of H_2_O_2_. This escalates the rate of enzyme inactivation leading to the cessation of the reaction. Therefore, in the next experiment, we doubled the enzyme concentration to 20 μM while maintaining all other conditions the same (Table 1, entry 2, Figure S2). Indeed, this resulted in a significantly more robust reaction with linear product accumulation (40.5 mM h^–1^ corresponding to >80% H_2_O_2_ yield) for at least 6 h.

This result encouraged us to increase the H_2_O_2_ feeding rate even more to 200 mM h^–1^ (Table 1, entry 3, Figure S3). Within 4 h, 373 mM product (332 mM cyclohexanol and 41 mM cyclohexanone) was formed corresponding to a productivity of 93 mM h^–1^ (9.3 g L^–1^ h^–1^). The reaction completely ceased after 4 h indicating complete inactivation of the biocatalyst. It is worth mentioning that in this experiment after completion, trace amounts (estimated less than 10 mM) of dual hydroxylation products (1,3- and 1,4-cyclohexanediol) were observed (Figure S8).

The reaction so far is limited by the still comparably low substrate concentration and the poor mass balance (mostly due to evaporation of the starting material), which in the current reactor setup is difficult to resolve. Initial attempts to address the loss of starting material in a fed-batch approach (Table 1, entry 4, Figure S4) largely failed as no improvement of the reaction robustness or overall product formation rate was achieved. Possibly, the occurrence of a liquid–liquid interface caused *Aae*UPO inactivation or reduced mass transfer. Further investigations aiming at understanding and resolving this issue are currently underway.

**Figure 2:**
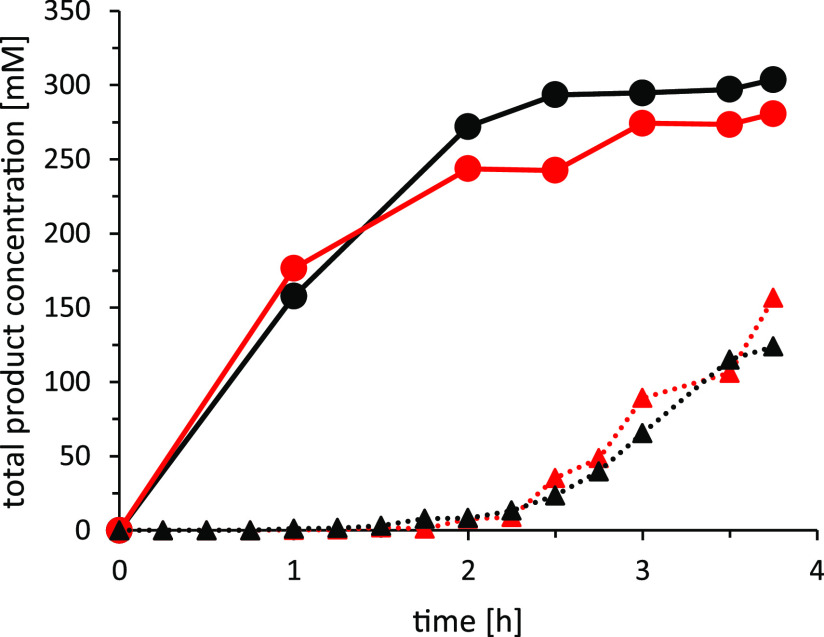
r*Aae*UPO-catalyzed oxidation of cyclohexane on a 10.9 L scale. Two individual batches are shown (black, red). (black circle, red circle): total product formed; (black triangle, red triangle): H_2_O_2_ concentration. Reaction conditions: 500 mM substrate (starting) concentration, 50 vol % ACN, 100 mM potassium phosphate buffer, pH 6.0, 20 μM *Aae*UPO (concentrated supernatant), H_2_O_2_ dosing rate: 120 mL h^–1^ from 12.75 M stock, (pump flow rate was 2 mL per min). The reaction was stirred at 225 rpm at 25 °C. The product concentration was calculated from calibration curves. GC analysis on achiral column (CP-Wax 52GB).

To demonstrate the scalability of the proposed *Aae*UPO-catalyzed oxyfunctionalization of cyclohexane, we performed two reactions with a working volume of 10.7 L in a 35 L reactor adopting the reaction conditions of Table 1, entry 3 (Figure 2). The average initial (1 h) product formation rate was at least 157 mM h^–1^ (approx. 15 g L^–1^ h^–1^). This corresponds well to the formal H_2_O_2_ addition rate (ca. 150 mM h^–1^) indicating that the H_2_O_2_ utilization was complete (close to 100% yield in H_2_O_2_). Hence, the H_2_O_2_ yield was significantly higher than in the smaller scale experiments (Table 1) with H_2_O_2_ yields ranging between 70 and 80%. In the latter case, the well-known catalase-activity of *Aae*UPO may account for the observation.^36^ Currently, we are lacking a plausible explanation for this upscaling effect. Interestingly within this initial period, practically only cyclohexanol was formed (cyclohexanol: cyclohexanone <25), whereas later cyclohexanone formation became more dominant (final ratio of cyclohexanol: cyclohexanone was approx. 10). However, the rate of product formation reduced in the next hour. As noted previously, this may be attributed to the depletion of the cyclohexane starting material (boiling point: 81 °C), which could not be quantified with our present analytical setup [due to similar boiling points of acetonitrile (boiling point: 82 °C) and the extraction solvent ethyl acetate (boiling point: 77 °C),^37^ separation of cyclohexane from the cosolvents on GC was not possible]. After 2 h, the product formation rate decreased considerably concomitant with the accumulation of H_2_O_2_.

Overall, 290 mM (average of two experiments) cyclohexanol/cyclohexanone was obtained corresponding to approx. 520 g of product and a yield of 58% (based on 500 mM initial starting material concentration). Hence, a product to catalyst ratio of 30.6 g_product_ g^–1^_*Aae*UPO_ can be estimated (TTN_*Aae*UPO_ = 13,000 mol mol^–1^).

Admittedly, the reaction presented here is not (yet) applicable for commercial-scale synthesis of the bulk chemical KA-oil. Significant improvements in process analytics (such as the quantification of the starting material and in situ H_2_O_2_ quantification to adjust the H_2_O_2_ dosing), substrate loading (e.g., by using two-liquid phase systems of ideally achieving solvent-free reaction conditions),^38−40^ and improving the catalyst usage (e.g., by further improving the H_2_O_2_-addition strategy) to minimize its cost contribution^41^ will be necessary to render the proposed biocatalytic oxyfunctionalization of cyclohexane industrially relevant. However, we are convinced that simple measures such as an adjusted ratio of H_2_O_2_ feed rate and *Aae*UPO concentration and in situ product removal^42^ will enhance the productivity and catalyst usage significantly. Furthermore, engineered variants of *Aae*UPO^43^ and suitable immobilization strategies^44^ will further improve the economic attractiveness.

It is, however, also worth mentioning that the oxyfunctionalization rates and product titers achieved in this study, to the best of our knowledge, surpass those reported for P450 MO-catalyzed pendants by orders of magnitude, thereby demonstrating the synthetic potential of the proposed peroxygenase technology.

## Conclusions

Overall, in this contribution, we have demonstrated the mol-scale biocatalytic hydroxylation of cyclohexane using the peroxygenase from *Agrocybe aegerita* (*Aae*UPO, PaDa-I). To the best of our knowledge, this is the first time an unspecific peroxygenase has been used at this scale. The promising results obtained in this preliminary study underscore the potential of peroxygenases as industrial catalysts.

## Supporting Information Available

*Aae*UPO-catalyzed oxidation of cyclohexane to cyclohexanol (blue) and cyclohexanone (gray) in 100 mL scale; qualitative tracking of H_2_O_2_ in the reaction mixture; calibration curve for cyclohexanol; calibration curve for cyclohexanone; and GCMS analysis of the *Aae*UPO-mediated conversion of cyclohexane after 6 h (PDF)
