# Supplementary material for: Toward Kilogram-Scale Peroxygenase-Catalyzed Oxyfunctionalization of Cyclohexane
Source: Org Process Res Dev. 2023 Jun 9;27(7):1384–9. doi: 10.1021/acs.oprd.3c00135 (PMC10367066; doi:10.1021/acs.oprd.3c00135)
Supplement: Supplementary file 1 — op3c00135_si_001.pdf [file op3c00135_si_001.pdf]

Towards Kilogram-scale Peroxygenase-catalyzed Oxyfunctionalization of Cyclohexane

*Thomas Hilberath,[1] Remco van Oosten, [1] Juliet Victoria, [2] Hugo Brasselet,[3] Miguel Alcalde, [4] John M. Woodley, [2] Frank Hollmann[1]**

[1] Department of Biotechnology, Delft University of Technology, van der Maasweg 9, 2629HZ, Delft, The Netherlands.

[2] Department of Chemical and Biochemical Engineering, Technical University of Denmark, 2800 Kgs. Lyngby, Denmark

[3] Holizymes B.V., van der Maasweg 9, 2629HZ, Delft, The Netherlands

[4] Department of Biocatalysis, Institute of Catalysis, CSIC, 28049 Madrid, Spain

* Corresponding author: Frank Hollmann

Email: f.hollmann@tudelft.nl

Supplementary information

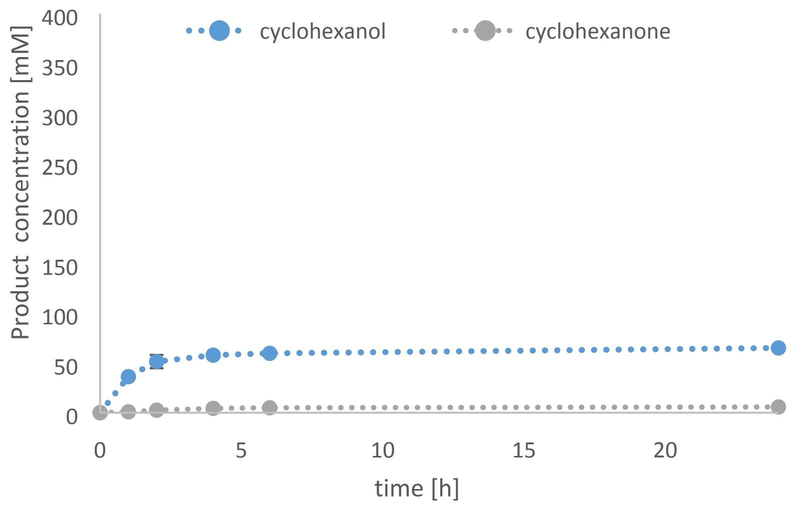

Figure S1: *Aae*UPO-catalyzed oxidation of cyclohexane to cyclohexanol (blue) and cyclohexanone (grey) in 100 mL scale. Reaction conditions: 500 mM substrate concentration, 100 mM potassium phosphate buffer, pH 6.0, 10 µM *Aae*UPO (concentrated supernatant), H_2O_2-dosing rate: 50 mM h^{-1}. The reaction was shaken at 300 rpm at 25°C. The product concentration was calculated from calibration curves and derived from two measured samples. GC-analysis on achiral column (CP-Wax 52GB).

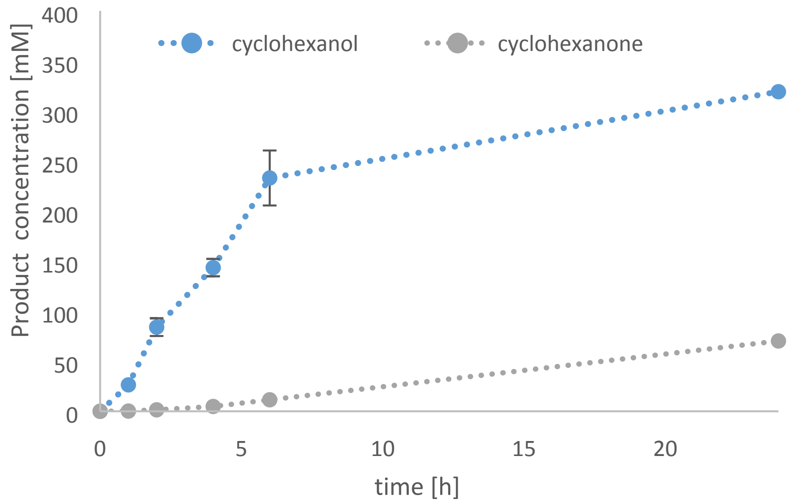

Figure S2: *Aae*UPO-catalyzed oxidation of cyclohexane to cyclohexanol (blue) and cyclohexanone (grey) in 100 mL scale. Reaction conditions: 500 mM substrate concentration, 100 mM potassium phosphate buffer, pH 6.0, 20 µM *Aae*UPO (concentrated supernatant), H_2O_2-dosing rate: 50 mM h^{-1}. The reaction was shaken at 300 rpm at 25°C. The product concentration was calculated from calibration curves and derived from two measured samples. GC-analysis on achiral column (CP-Wax 52GB).

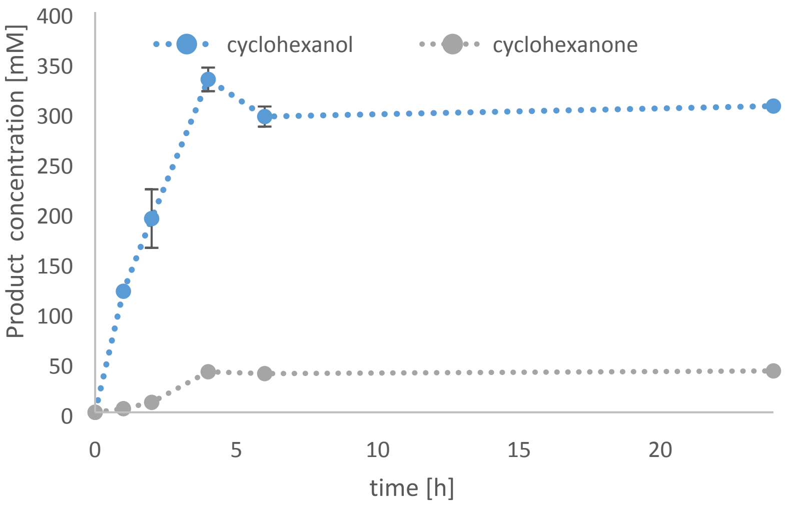

Figure S3: *Aae*UPO-catalyzed oxidation of cyclohexane to cyclohexanol (blue) and cyclohexanone (grey) in 100 mL scale. Reaction conditions: 500 mM substrate concentration, 100 mM potassium phosphate buffer, pH 6.0, 20 µM *Aae*UPO (concentrated supernatant), H_2O_2-dosing rate: 200 mM h^{-1}. The reaction was shaken at 300 rpm at 25°C. The product concentration was calculated from calibration curves and derived from two measured samples. GC-analysis on achiral column (CP-Wax 52GB).

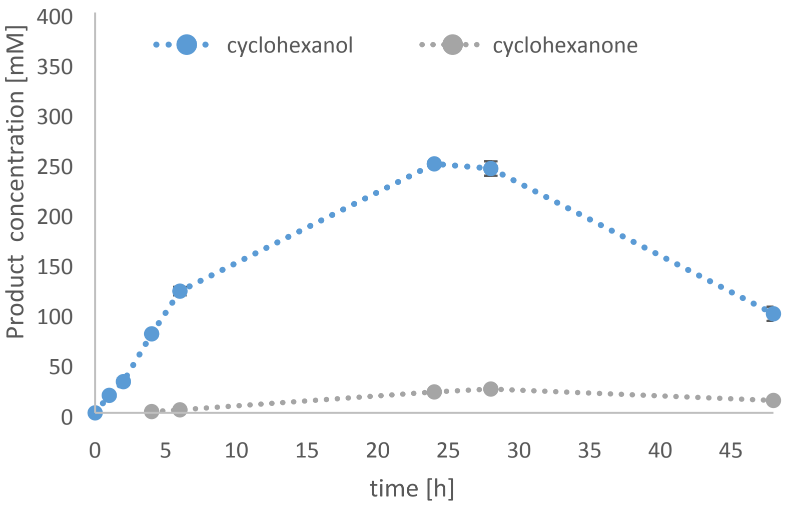

Figure S4: *Aae*UPO-catalyzed oxidation of cyclohexane to cyclohexanol (blue) and cyclohexanone (grey) in 100 mL scale. Reaction conditions: 500 mM substrate concentration, 100 mM potassium phosphate buffer, pH 6.0, 20 µM *Aae*UPO (concentrated supernatant), H_2O_2-dosing rate: 200 mM h^{-1}. The reaction was shaken at 300 rpm at 25°C. After 6 and 28 h, 2.7 mL of substrate were added. The product concentration was calculated from calibration curves and derived from two measured samples. GC-analysis on achiral column (CP-Wax 52GB).

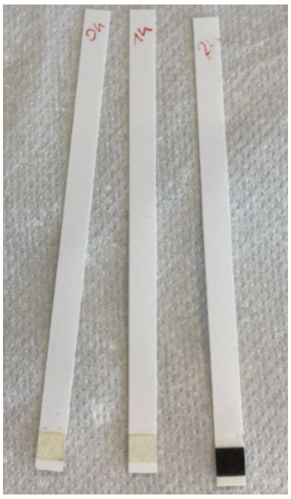

Figure S5: Qualitative tracking of H_2O_2 in the reaction mixture. A blue color indicates presence of H_2O_2.

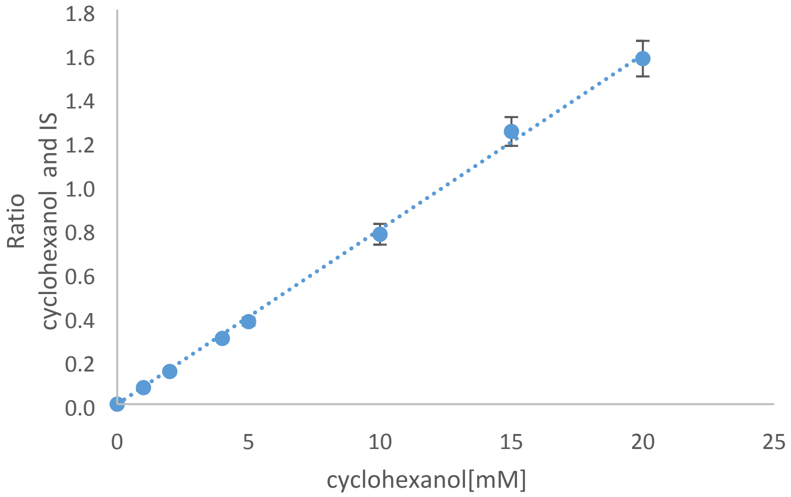

Figure S6: Calibration curve for cyclohexanol. 0 – 20 mM cyclohexanol in 10 vol% acetonitrile were prepared in 0.5 mL 100 mM potassium phosphate buffer, pH 6 and extracted with an equal volume of ethyl acetate containing 5 mM of the internal standard *n*-dodecane.

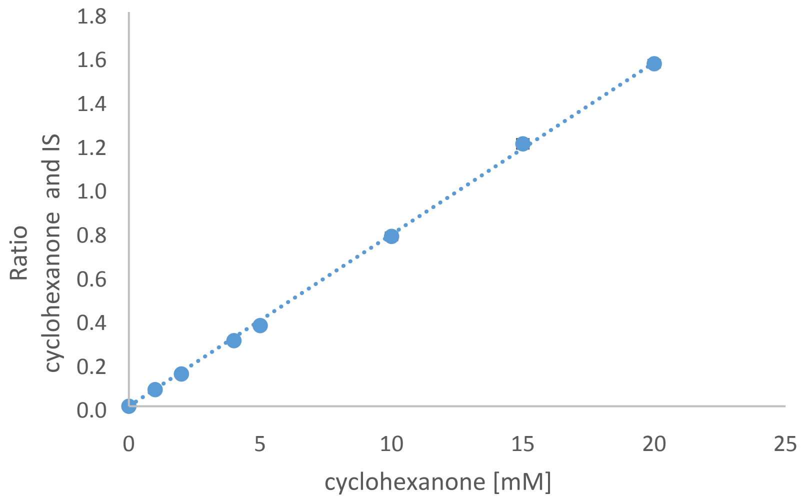

Figure S7: Calibration curve for cyclohexanone. 0 – 20 mM cyclohexanone in 10 vol% acetonitrile were prepared in 0.5 mL 100 mM potassium phosphate buffer, pH 6 and extracted with an equal volume of ethyl acetate containing 5 mM of the internal standard *n*-dodecane.

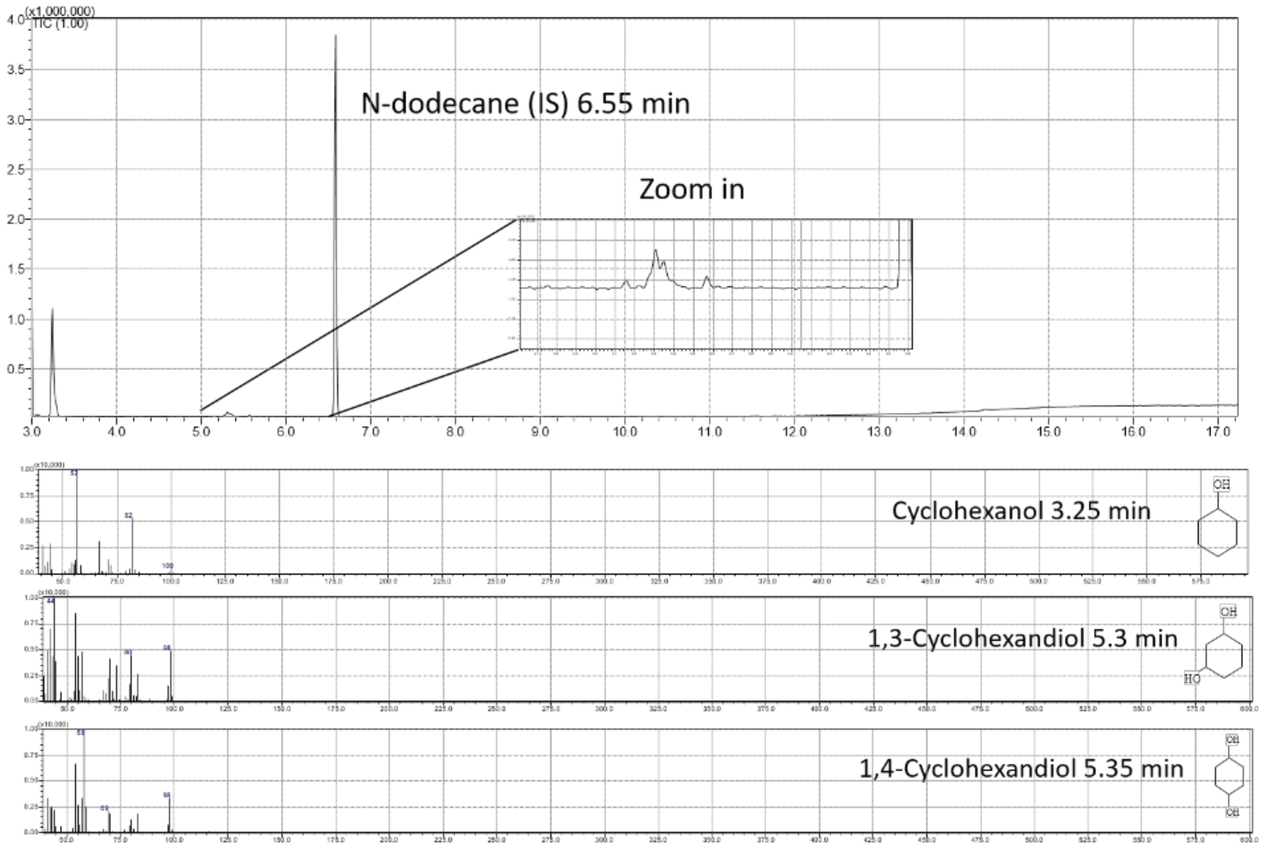

Figure S8: GCMS analysis of the *Aae*UPO-mediated conversion of cyclohexane after 6 h (experiment 3). Cyclohexanol and respective diols were identified by their fragmentation pattern using the NIST-database.
